# Internet Gaming Disorder Increases Mind-Wandering in Young Adults

**DOI:** 10.3389/fpsyg.2020.619072

**Published:** 2021-01-28

**Authors:** Jiawen Zhang, Hui Zhou, Fengji Geng, Xiaolan Song, Yuzheng Hu

**Affiliations:** ^1^Department of Psychology and Behavioral Sciences, Zhejiang University, Hangzhou, China; ^2^College of Education, Zhejiang University, Hanghzou, China; ^3^College of Teacher Education, Zhejiang Normal University, Jinhua, China

**Keywords:** Internet gaming disorder, social anxiety, mind-wandering, mediator effect, behavioral addiction

## Abstract

As a primary symptom defining Internet gaming disorder (IGD), preoccupation indicates a mind state in which gamers think about a gaming activity so much that other things appear less important and/or interesting to them. Previous studies have examined the negative impacts of IGD on both cognitive and affective functions, yet no study has investigated the influence of IGD on daily mind state changes that interfere with ongoing tasks. The current study hypothesized that more IGD symptoms lead to a higher frequency of mind state shift in terms of mind-wandering. As social anxiety is related to both IGD and mind-wandering, we further hypothesized that social anxiety would partially contribute to the relationship. Survey data were collected from 632 young adults who were divided into two groups based on whether they reported playing games or not. In the player group, the number of IGD symptoms present was positively related to mind-wandering (*r* = 0.269, *p* < 0.001) and social anxiety (*r* = 0.235, *p* < 0.001), with the latter two showing a positive correlation in both players (*r* = 0.37, *p* < 0.001) and non-players (*r* = 0.57, *p* < 0.001). A mediation analysis for the players showed a partial mediation effect of social anxiety on the relationship between IGD and mind-wandering (mediation effect: PM = 0.292, *p* < 0.001), and the model was replicated in an independent sample. This study suggests that excessive gaming behavior may increase mind-wandering and a shift towards such a non-productive mind state could exert long-term detrimental effects in adolescents and young adults.

## Introduction

The popularity of Internet games has profound impacts on human society and individuals in many ways. The impact of the high prevalence of Internet gaming especially on young people is indispensable (Wang et al., 2014). In contrast to the real physical world we live in, some video games create virtual worlds with functional objects and/or characters that are mentally compelling to most people. Because it is so attractive to some individuals, they repeatedly spend too much time on playing games regardless of negative consequences, raising the public concern of Internet gaming disorder (IGD). To address the increasing public concern regarding the negative consequences of excessive digital gaming, official bodies, including the American Psychiatric Association (2013), have suggested the unification and consensus of IGD. Later, Petry et al. (2014) proposed nine diagnostic items for IGD, corresponding to the nine DSM-5 constructs including preoccupation, tolerance, withdrawal, persistence escape, problems, deception, displacement, and conflict (American Psychiatric Association, 2013). The total number of these nine symptoms’ presence during the past 12 months is used to characterize IGD severity.

As a new phenomenon in the digital era, both the risk factors and the negative effects of IGD are interesting topics of ongoing research. A number of studies have shown that the quality of parent–child relationships may be the primary risk factors of IGD (Sugaya et al., 2019). In contrast, IGD is also related to cognitive and psychosocial problems such as impaired executive control (Dong et al., 2010), higher loneliness (Qin et al., 2007), lower self-esteem (Lemmens et al., 2011), and social phobia (Sioni et al., 2017). Although preoccupation with gaming has been listed as one primary diagnostic symptom in DSM-5, these previous studies have only examined the negative impacts of IGD on cognitive and affective functions. Preoccupation with gaming activity indicates a mind state in which gamers think about a gaming activity so much that other things appear less important and/or interesting to them. A shift towards such a non-productive mind state could exert long-term detrimental effects in adolescents and young adults who need to devote tremendous time in study to equip them well for the rest of their life. To our best knowledge, no study so far has investigated the influence of IGD on daily mind state shift.

The shift of mind state away from an ongoing task is usually termed as mind-wandering (MW; Smallwood and Schooler, 2006). It has been demonstrated that mind-wandering occupies as much as 30–50% of our waking life (Kane et al., 2007; Schooler et al., 2011), and mind-wandering is even regarded as the brain’s default mode of operation because of its spontaneous feature (Raichle et al., 2001; Buckner et al., 2008; Christoff et al., 2009; Baird et al., 2012). Thereby, studies of the neural underpins of mind-wandering have primarily focused on the default mode network that is metabolically more active at resting state than the task state when attention is oriented towards an external stimulus (Buckner et al., 2008). The level of mind-wandering has been shown to associate with the default mode network activity (Mason et al., 2007). Although it seems a trait-like personal character, previous studies have demonstrated that the level of mind-wandering can be reduced through mindful training (Rahl et al., 2016), suggesting that it can be changed in response to behavioral adaptation. Hypothetically, it also could be altered by other activities, such as excessive gaming, that leads to the emergence of a preoccupation symptom of IGD. Although mind-wandering may not always associate with negative outcomes (Baird et al., 2012), a large body of research has found that a high level of mind-wandering is related to negative cognitive and emotional consequences. For example, mind-wandering is regarded as a pervasive and problematic influence on the performance and well-being of adolescents (Mrazek et al., 2013b). Research has also found that the problems of mind-wandering among young people are widespread and significant (Polderman et al., 2010). The frequency of mind-wandering during lectures is specifically related to worse learning, and high-level mind-wandering leads to diminishing reading comprehension in college students (Risko et al., 2012). In addition to the cognitive aspects, literature has revealed that mind-wandering is associated with worse mood, less self-esteem, lower satisfaction, and more perceived stress (Killingsworth and Gilbert, 2010; Mrazek et al., 2013b), which, on the other hand, are closely related to social activity anxiety (Argyle and Lu, 1990; Neto, 1993; van Tuijl et al., 2014). A study found that patients with social anxiety disorder had high attention deficit–hyperactivity disorder (ADHD) comorbidity (Koyuncu et al., 2015), suggesting the positive correlation between social anxiety and mind-wandering resulting from attention failure. Another study showed that anxiety played an important role in MW in both ADHD and non-ADHD individuals (Figueiredo, 2020). The correlation between anxiety and mind-wandering has also been supported by neuropsychological studies on affective dysfunction and attention failure (Seli, 2019). Although there are no studies showing a direct relation between social anxiety (SA) and MW, one would speculate based on the above-mentioned studies that a high level of social anxiety is associated with more frequent mind-wandering.

Studies have shown that social anxiety plays an important role in the development of gaming disorders (Vanzoelen and Caltabiano, 2016). Individuals who feel anxious and have difficulty building relationships in the real world may choose the Internet to alleviate such anxiety. Online gaming is an ideal platform for individuals with high levels of social anxiety to build interpersonal relationships in which they can establish personal factions, organizations, and teams (Brenner, 1982). On the other hand, as time spent playing online games increases, the quality of interpersonal relationships decreases and the level of social anxiety increases (Lo et al., 2005), suggesting an interaction between online gaming disorder and social anxiety. Although online games may temporarily alleviate social anxiety, they do not improve real-world social relationships. On the contrary, the satisfaction of online games can encourage people to overindulge in the virtual world, which can lead to impairment of real-world relationships.

The current study, therefore, aims to examine whether IGD is associated with a high level of mind-wandering and how social anxiety plays a role in this relationship.

In summary, the preoccupation with gaming in IGD may lead to a high frequency of task disruption that defines mind-wandering. Social anxiety concurrent with IGD may also exert an impact on mind-wandering as anxiety has also been shown to interfere with task-focused thinking (Sarason, 1984), yet no empirical evidence has been shown to articulate such relationships. The objective of the present study was to examine the relationships among IGD, social anxiety, and mind-wandering. We hypothesize that (1) IGD is positively associated with the level of mind-wandering, (2) IGD is positively related to the level of social anxiety, and (3) the relationship between IGD and mind-wandering is partially mediated by social anxiety.

## Materials and Methods

### Subjects and Procedures

The participants were asked to fill out an online survey concerning Internet gaming activity, social anxiety, mind-wandering, and demographic information (see section “Measures”). If the respondents reported that they play Internet games, they were asked to report the average time spent on Internet games per day during the past 12 months. Four catch trials were pseudorandomly distributed into the survey to identify the participants who fulfilled the survey carelessly. The respondents were assured in the survey instruction that this information would be used for the purpose of scientific research and would be analyzed only by the research team. In total, 687 young people (35% male) participated, and they each received three RMB for compensation of their time. The participants who did not answer the catch trials correctly or reported “do not play game” but responded to one or more IGD symptoms were excluded from analysis. Finally, 637 respondents (93%) were included in the statistical analyses (see section “Statistic Analysis”). We call this cohort “the primary sample” to differentiate it from the replication sample (see section “Replication”).

### Measures

#### IGD Symptoms Based on DSM-5

The Chinese version of the nine-item scale for IGD developed based on DSM-5, which was referred to as the DSM scale in the present study, was used to characterize gaming activity-associated addiction-like behaviors (Petry et al., 2014). These items were proposed by international experts to measure the intended concept behind each of the nine DSM-5 criteria for IGD and were translated into 10 main languages including Chinese (Petry et al., 2014). This scale includes one item for each of the nine underlying IGD diagnostic criteria. According to DSM-5, tentative gaming disorder is identified based on the total number of the nine symptoms met in the past 12 months (American Psychiatric Association, 2013). The respondents rated all items with either no (0) or yes (1), yielding DSM scores ranging from 0 to 9. The DSM scale had a Cronbach’s alpha of 0.74 in the present study.

#### Social Anxiety

Social anxiety was measured using the Chinese version of Liebowitz Social Anxiety Scale (LSAS; Liebowitz and Klein, 1987; He and Zhang, 2004). The LSAS (Liebowitz and Klein, 1987) comprises 24 social situations to be rated for the level of fear (0 = none to 3 = severe) as well as avoidance (0 = none to 3 = usually) for the past week. The LSAS has good psychometric properties in European American samples (Heimberg et al., 1999), and the Chinese version of LSAS also shows excellent internal consistency and temporal stability (He and Zhang, 2004). The LSAS score is calculated by the sum of the fear and avoidance ratings of the 24 items, yielding a score range of 0 to 144 (i.e., 3 × 24 + 3 × 24), with a higher score indicating a high level of social anxiety. The LSAS questionnaire had a Cronbach’s alpha of 0.96 in the present study.

#### Mind-Wandering

Mind-wandering was characterized using the Mind-Wandering Questionnaire (MWQ) (Mrazek et al., 2013b). The MWQ is a five-item questionnaire that evaluates the levels of mind-wandering trait with a six-point Likert-type scale ranging from 1 (almost never) to 6 (almost always). The total MWQ score is calculated by the sum of the five items, yielding a score range of 5–30, with a higher score indicating a higher frequency of mind-wandering. The Chinese version of the MWQ has been validated as good, with a Cronbach’s alpha of 0.72 in a previous study (Luo et al., 2016). In the current study, it had a Cronbach’s alpha of 0.84.

### Statistical Analysis

The subjects were grouped into non-game player and game player subgroups based on the self-report of game playing, and the demographic information was summarized for each subgroup separately. Then, we tested the three hypotheses about the relationships among IGD, social anxiety, and mind-wandering in the game player subgroup with correlational analyses and mediation analyses correspondingly. We also conducted a complementary analysis to examine the differences in mind-wandering and social anxiety between non-game players and three subgroups of game players divided based on DSM-5 symptoms present in them. All the statistical analyses were conducted with SPSS 25.0. The mediation analyses were carried out using the SPSS macro PROCESS (model 4)^1^ as suggested by Hayes (2017). To test the mediation model in which we hypothesized that social anxiety (LASA score, the mediator variable *M*) mediates the relationship between mind-wandering (MWQ score, the outcome variable *Y*) and IGD (DSM score, the independent variable *X*), the PROCESS estimates the following three regression models (with covariates omitted).

$$\mathrm{The}\,\mathrm{mediator}\,\mathrm{model}:M=i_{1}+a\,X+e_{1}$$

$$\mathrm{The}\,\mathrm{conditioned}\,\mathrm{model}:Y=i_{2}+b\,M+c^{\prime }\,X+e_{2}$$

$$\mathrm{The}\,\mathrm{total}\,\mathrm{effect}\,\mathrm{model}:Y=i_{3}+c\,X+e_{3}$$

where *i*_1_, *i*_2_, and *i*_3_ are regression intercepts, *e*_1_, *e*_2_, and *e*_3_ are errors in the estimation of each model, respectively, and *a*, *b*, *c*, and *c*′ are the regression coefficients. The indirect effect of IGD (*X*) on mind-wandering (*Y*) through social anxiety (*M*) is the product of *a* and *b*, and the significance of this indirect effect is indicated by the bootstrap confidence intervals such that if the interval does not across zeros, the mediation effect is considered as significant (Hayes, 2017). The direct effect of IGD (*X*) on mind-wandering (*Y*) is indicated by *c*′, and the significance of *c*′ involves testing the null hypothesis *c*′ = 0.

### Replication

To validate our mediation model, we conducted another survey in an independent sample. In addition to the DSM scale used in the initial survey, Internet Addiction Test (IAT; Young and De Abreu, 2010) was also included in the replication survey to assess the robustness of the mediation model. The IAT is a 20-item questionnaire concerning Internet use-associated problematic behaviors, including psychological dependence, compulsive use, and withdrawal as well as problems related to school, sleep, family, and time management. All items were scored on a five-point Likert-scale (never, rarely, occasionally, often, and always, corresponding to scores from 1 to 5). The IAT has been validated as a reliable self-report instrument that can be used to characterize IGD (Widyanto et al., 2010). In total, data from 276 respondents were collected, and 35 were excluded from the analysis based on the same exclusion criteria used in the primary sample. Finally, 181 respondents who reported to play games were used to assess the reproducibility of the model, with DSM score and IAT-20 score as the independent variables (*X*) separately.

## Results

### Descriptive Statistics of the Primary Sample

In the primary sample, the subjects’ age ranged between 17 and 30 years (*M* = 20.7, SD = 1.9). Based on the answer to the question “Do you play Internet game? Yes/No,” 84 (12% male) subjects who responded “No” were grouped into the non-game player group, whereas the rest (553, 40%, male) of the subjects who responded “Yes” were grouped into the game player group. Among the game players, 73% (*N* = 408) reported playing Internet games for less than 2 h per day, 20% (*N* = 108) reported playing Internet games for 2–4 h per day, and 7% (*N* = 37) reported playing Internet games for more than 4 h per day on average in the last 12 months.

### Correlations Among IGD, Mind-Wandering, and Social Anxiety in Game Players

**FIGURE 1:**
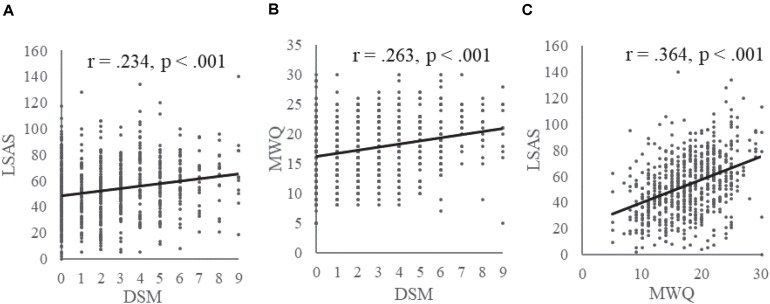
Correlations between Internet gaming disorder symptoms, social anxiety, and mind-wandering. The DSM symptoms were positively correlated with social anxiety as measured by Liebowitz Social Anxiety Scale **(A)** and mind-wandering **(B)**, with the latter two also positively correlated with each other **(C)**.

In the game player group, DSM score was significantly and positively correlated with social anxiety as measured by LSAS (*r* = 0.234, *p* < 0.001, Figure 1A) and mind-wandering (*r* = 0.263, *p* < 0.001, Figure 1B), with the latter two constructs also significantly and positively correlated (*r* = 0.364, *p* < 0.001, Figure 1C). No gender effect was found in the three measurements, while age was positively correlated with DSM score (*r* = 0.469, *p* < 0.001).

### The Mediation Role of Social Anxiety on the Relationship Between IGD and Mind-Wandering

The predicted mediation model was tested with Andrew F. Hayes’ SPSS macro PROCESS (Hayes, 2017) by evaluating the three regression models described above, with the resulting coefficients and statistics listed in Table 1. As shown by the mediator model (*F* = 11.279, *R*^2^ = 0.058, *p* < 0.001), conditioned model (*F* = 28.559, *R*^2^ = 0.173, *p* < 0.001), and total effect model (*F* = 15.655, *R*^2^ = 0.079, *p* < .001), the effect on mind-wandering attributed to IGD (the DSM score) was reduced from 0.298 to 0.216 (see Table 1) by social anxiety, the mediator variable. The significance of this indirect effect of IGD on mind-wandering through social anxiety was bootstrapped using 5,000 bootstrap samples with replacement. The point estimate of the indirect effect was 0.082, with a standard error of 0.018. The 95% bias-corrected confidence interval (from 0.049 to 0.121) did not include zeros, indicating that the mediation effect was significant. In addition, the direct effect of IGD, *c*′, remained significant in the conditioned model (*c*′ = 0.216, *p* < 0.001, Table 2), indicating a partial rather than complete mediation by social anxiety. Given that the total effect of IGD on mind-wandering was 0.298 and the indirect effect through social anxiety was 0.082, social anxiety accounted for 27.5% of the effect of IGD on mind-wandering.

**TABLE 1:** Effects in the mediation model.

Model (outcome variable)		*β*	SE	*T*	*P*
Mediator (Liebowitz Social Anxiety Scale, LSAS)	Constant	61.153	11.071	5.524	<0.001
	DSM	0.261	0.461	5.568	<0.001
	Gender	–0.021	1.954	–0.502	0.616
	Age	–0.061	0.548	–1.296	0.195
Conditioned (MWQ)	Constant	16.463	2.344	7.024	<0.001
	LSAS	0.315	0.009	7.877	<0.001
	DSM	0.216	0.089	4.776	<0.001
	Gender	–0.058	0.403	–1.495	0.136
	Age	–0.064	0.113	–1.443	0.150
Total effect (MWQ)	Constant	20.699	2.405	8.607	<0.001
	DSM	0.298	0.100	6.432	<0.001
	Gender	–0.065	0.424	–1.579	0.164
	Age	–0.083	0.119	–1.784	0.021

		**Effect**	**BootSE**	**BootLLCI**	**BootULCI**

Indirect effect		0.082	0.018	0.049	0.121

**TABLE 2:** Effects in the mediation model (DSM as independent variable).

Model (outcome variable)	*F*	*r*^2^	*p*		*β*	SE	*t*	*P*
Mediator (Liebowitz Social Anxiety Scale, LSAS)	6.578	0.100	<0.001	Constant	61.425	8.264	7.433	<0.001
				DSM	0.249	0.394	3.491	<0.001
				Gender	0.190	1.828	2.656	0.009
				Age	–0.065	0.381	–0.906	0.366
Conditioned (MWQ)	15.953	0.266	<0.001	Constant	13.171	3.138	4.198	<0.001
				DSM	0.205	0.135	3.067	0.003
				LSAS	0.397	0.025	5.827	<0.001
				Gender	0.054	0.618	0.815	0.416
				Age	–0.101	0.126	–0.156	0.121
Total effect (MWQ)	8.390	0.124	<0.001	Constant	22.089	2.983	7.404	<0.001
				DSM	0.304	0.142	4.315	<0.001
				Gender	0.130	0.660	1.832	0.069
				Age	–0.127	0.137	–1.797	0.074

					**Effect**	**BootSE**	**BootLLCI**	**BootULCI**

Indirect effect					0.099	0.033	0.037	0.168

### Complementary Results Comparing Game Players vs Non-game Players

To compare the differences in mind-wandering and the level of social anxiety between non-gamers and game players with different IGD severity, the game players were assigned to the following four subgroups according to Lemmens et al. (2015): (1) low-risky game players (LG) that included those with DSM score lower than 3 (*n* = 202, MWQ = 16.41 ± 4.88; LSAS = 48.00 ± 22.318), (2) risky game players (RG) that included those with DSM score between 3 and 5 (*n* = 267, MWQ = 18.55 ± 4.67; LSAS = 56.50 ± 22.04), (3) high-risky game players (HG) that included those with DSM score higher than 5 (*n* = 84, MWQ = 19.85 ± 4.976; LSAS = 61.63 ± 21.78), and (4) non-game players (NG) who reported not playing online games (*n* = 84, MWQ = 16.77 ± 5.82; LSAS = 53.40 ± 24.27).

**FIGURE 2:**
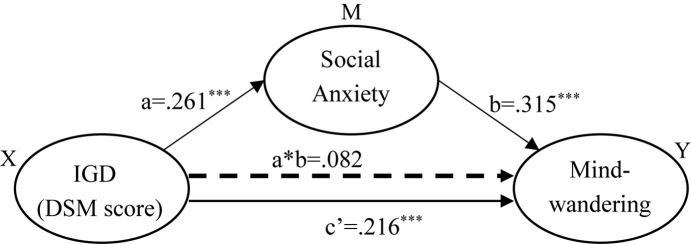
The average scores of Mind-Wandering Questionnaire (MWQ) and Liebowitz Social Anxiety Scale (LSAS) in different subgroups. ANOVAs and *post hoc* tests with multiple comparisons were conducted to compare the difference between non-gamers (NG) and three subgroups of gamers. The MWQ score in NG were significantly lower than in risky game players (*p* = 0.006) and risky game players (*p* < 0.001). The LSAS score in NG were not significantly different than those in the game groups. ^∗^*p* < 0.05, ^∗∗^*p* < 0.01, ^∗∗∗^*p* < 0.001.

Analysis of variances (ANOVAs) and post-test with Bonferroni correction were run to test the differences of MWQ and LSAS score between NG and each player subgroup (LG, RG, and HG). The result showed significant group effects on MW (*F* = 10.21, *p* < 0.001) and SA (*F* = 14.11, *p* < 0.001). The result of the *post hoc* tests with multiple comparisons showed significantly higher MWQ scores in the HG group than those in the NG group (*p* < 0.001). The MWQ scores were also significantly higher in the RG group than those in the NG group (*p* = 0.006). Regarding the difference in LSAS score, no differences were shown between NG and other player groups. Both MWQ and LSAS scores in NG showed no significant difference from those in the LG group (Figures 2A,B).

### Replication of the Mediation Model

The replication sample has an average age of 21 years, with a standard error of 2.7 years, and 95 (52%) out of the total 181 respondents were female. The Cronbach’s alpha of DSM (*M* = 3.49, SE = 2.32), IAT-20 (*M* = 55.68, SE = 13.91), LSAS (*M* = 61.57, SE = 12.78), and MWQ (*M* = 19.71, SE = 4.68) were 0.716, 0.915, 0.925, and 0.806, respectively. The four variables were positively correlated with each other (Table 3).

**TABLE 3:** Correlations between DSM, IAT-20, Liebowitz Social Anxiety Scale (LSAS), and Mind-Wandering Questionnaire (MWQ) in the replication sample.

	IAT-20	LSAS	MWQ
DSM	0.493***	0.240***	0.298***
IAT-20	–	0.499***	0.528***
LSAS	–	–	0.240***

***p < .001.

#### Mediation Model With DSM Score as Independent Variable

**FIGURE 3:**
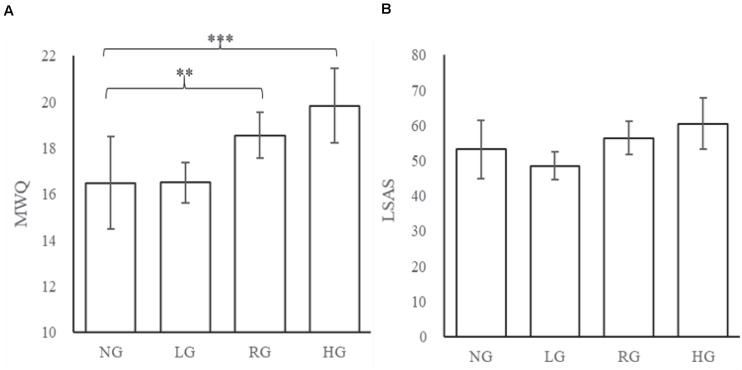
Mediation analysis (DSM as independent variable) in the replication sample shows that the relationship between Internet gaming disorder and mind-wandering is partially mediated by social anxiety. ^∗∗^*p* < 0.01, ^∗∗∗^*p* < 0.001.

The parameter estimations of the three models are shown in Table 2. As shown in the table, the effect on mind-wandering attributed to IGD (DSM score) was reduced from 0.304 to 0.205 by the mediator variable social anxiety (LSAS). Bootstrapping with 5,000 bootstrap samples estimated an indirect effect of social anxiety to be 0.100, with a standard error of 0.034 and a 95% bias-corrected confidence interval from 0.035 to 0.169. In addition, the direct effect of IGD, *c*′, also remained significant in the conditioned model (*c*′ = 0.205, *p* = 0.003, Table 2), indicating a partial rather than complete mediation by social anxiety (Figure 3). Social anxiety accounted for 32.6% effect of IGD on mind-wandering in the replication sample.

#### Mediation Model With IAT-20 Score as Independent Variable

**FIGURE 4:**
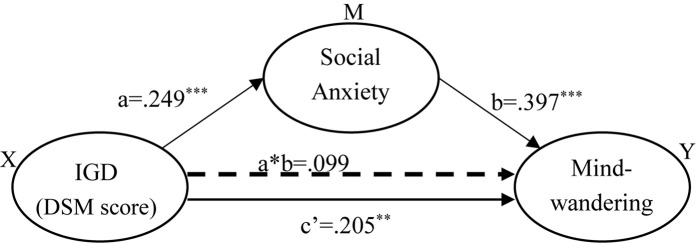
Mediation analysis (IAT-20 as independent variable) in the replication sample shows that the relationship between Internet gaming disorder and mind-wandering is partially mediated by social anxiety. ^∗∗^*p* < 0.01, ^∗∗∗^*p* < 0.001.

The significance tests of the three models with IAT-20 indicating IGD severity are shown in Table 4. As shown by the statistical parameters of the three models, the effect on mind-wandering attributed to IGD (IAT-20) was reduced from 0.517 to 0.387 by the mediator variable social anxiety (LSAS). The bootstrapped indirect effect was 0.100, with a standard error of 0.130 and a 95% bias-corrected confidence interval from 0.056 to 0.212. In addition, the direct effect of IGD, *c*′, also remained significant in the conditioned model (*c*′ = 0.387, *p* < 0.001, Table 4), indicating a partial rather than complete mediation by social anxiety (Figure 4). Social anxiety accounted for 25.1% effect of IGD on mind-wandering when IGD was characterized with IAT-20.

**TABLE 4:** Effects in the mediation model (IAT-20 as independent variable).

Model (outcome variable)	*F*	*r*^2^	*p*		*β*	SE	*t*	*P*
Mediator (Liebowitz Social Anxiety Scale, LSAS)	20.276	0.256	<0.001	Constant	34.778	8.615	4.037	<0.001
				IAT-20	0.483	0.062	7.191	<0.001
				Gender	0.083	1.696	1.242	0.216
				Age	0.009	0.351	0.134	0.894
Conditioned (MWQ)	22.155	0.335	<0.001	Constant	8.518	3.124	2.727	0.007
				IAT-20	0.387	0.024	5.347	<0.001
				LSAS	0.269	0.026	3.769	0.002
				Gender	–0.010	0.591	–0.155	0.877
				Age	–0.051	0.122	–0.810	0.419
Total effect (MWQ)	23.083	0.281	<0.001	Constant	11.936	3.099	3.852	<0.001
				IAT-20	0.517	0.022	7.828	<0.001
				Gender	0.012	0.610	0.190	0.850
				Age	–0.048	0.126	–0.745	0.457

					**Effect**	**BootSE**	**BootLLCI**	**BootULCI**

Indirect effect					0.13	0.040	0.056	0.2177

## Discussion

**FIGURE 5:**
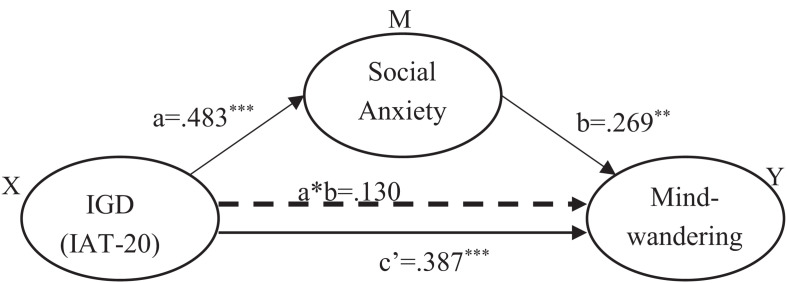
Mediation analysis showing that the relationship between Internet gaming disorder and mind-wandering is partially mediated by social anxiety. ^∗∗∗^*p* < 0.001.

In the present study, associations among IGD, mind-wandering, and social anxiety were found to be positive and significant (Figure 1). Furthermore, we found that the positive relationship between gaming disorder and mind-wandering was partly mediated by social anxiety (Figure 5). These relationships were replicated in an independent sample (Figures 3, 4). Comparing with non-players, only high-risky gaming players showed higher social anxiety, but both risky and high risky players showed a significantly high frequency of mind-wandering (Figure 2). Previous studies on gaming disorder have mostly focused on the cognitive and affective aspects of IGD (Mehroof and Griffiths, 2010; King and Delfabbro, 2014; Ko et al., 2014; Bargeron and Hormes, 2017; Yen et al., 2017). The present study suggests that mind-wandering, which is defined as task-irrelevant thoughts (Smallwood and Schooler, 2006), is a new dimension of IGD-related behavior.

The DSM-5 includes a preoccupation criterion reflecting how often an individual consistently thinks about gaming during non-gaming time. Based on this diagnostic symptom, pathological gaming activity may increase the frequency of mind-wandering when game players are not playing Internet games, and the current study provides empirical evidence for this speculation. Previous neural imaging studies have also provided evidence for the potential correlation between IGD and mind-wandering. For example, a fMRI study scanning adolescents with IGD under resting state showed altered connectivity in the default mode network (Ding et al., 2013). Specifically, the connectivity with the PCC was positively correlated with Internet addiction scores in the posterior cingulate gyrus (Ding et al., 2013), suggesting that participants with higher Internet addiction scores had increased activity in the DMN at resting state, which is possibly related to a higher level of mind-wandering. In the following paragraphs, we discuss how elevated mind-wandering may exert a negative effect on individuals with excessive gaming activity.

Mind-wandering will interrupt with ongoing task and impair task performance. Although why IGD severity is positively correlated with mind-wandering is elusive, a possible route could be that excessive gaming behavior alters the attention system or simply taps on the default mode network, resulting in higher-level mind-wandering. Regardless of the causality, more mind-wandering concurrent with IGD may cause diverse problems relating to academic performance and daily life. For example, high-level mind-wandering leads to a worse mood (Killingsworth and Gilbert, 2010) and significant academical performance costs (Wammes et al., 2016). Mind-wandering has also been found to natively predict learning (post-test) outcomes (Kane et al., 2017). To discourage task-irrelevant mind-wandering, interpolating lectures with memory tests can help students keep their attention to the lecture content, and task-relevant activities such as note-taking are also helpful to reduce mind-wandering and improve learning (Szpunar et al., 2013). In addition, cultivating mindfulness meditation training is also suggested as an effective and efficient technique for improving working memory capacity and academic performance by reducing mind-wandering (Mrazek et al., 2013a). The present study, together with the above-mentioned studies, suggests that mind-wandering could be a detrimental psychological effect in IGD.

A recent study has also recognized the importance to distinguish between deliberate and spontaneous subtypes of mind-wandering (Seli et al., 2015). The former involves the engagement of controlled processes for internal processing, whereas the latter reflects a failure of executive control (Seli et al., 2015). Elevated mind-wandering associated with pathological gaming behavior could originate from both types of mind-wandering. On one hand, individuals with excessive Internet gaming behavior may be more likely to crave for gaming when they are not playing games, which could be regarded as deliberate mind-wandering. On the other hand, one of the criteria of IGD, withdrawal, refers to symptoms that emerge when unable to play or attempting to cut down or stop gaming. The symptoms typically involve feeling restless, irritated, angry, frustrated, anxious, or sad, which suggest that excessive Internet gaming may increase the level of spontaneous mind-wandering by impacting the self-regulation system. Further studies are required to distinguish between the impacts of excessive gaming behavior on the two subtypes of mind-wandering. Nevertheless, either way exerts negative impacts on an individual’s long-term benefits.

A previous study has shown an association between excessive Internet use and poor emotional and social skills for social adaptation (Engelberg and Sjöberg, 2004). A higher social anxiety level in the IGD population has also been reported by previous research (Yen et al., 2007). There is also supporting evidence for a positive correlation between IGD and shyness (Lavin et al., 2004), loneliness (Ceyhan and Ceyhan, 2009), and avoiding social relationships (Kraut et al., 1998). The results of the current study are in line with these previous findings showing the co-occurrence of Internet addiction and social anxiety (Lo et al., 2005; Vanzoelen and Caltabiano, 2016). Although a 2-year prospective study showed that social phobia predicted Internet addiction among female individuals, male adolescents were not vulnerable to the effects of depression and social phobia on Internet addiction (Ko et al., 2009). In contrast, Lemmens et al. (2011) revealed that loneliness was a consequence of pathological gaming, which suggested that pathological gaming behavior may deteriorate existing interpersonal relationships and increase gamers’ loneliness. It is also possible that the level of social anxiety would increase with such increased feeling of loneliness. In addition, Strittmatter et al. (2015) found that gamers with pathological Internet use showed more peer problems. A huge amount of time spent on gaming might lead to deprivation of interaction with people in real life. They might feel less competent in social relationships and worried about their social abilities more often, resulting in more occurrences of mind-wandering. Most importantly, the ever-raising concern and the diagnosis of IGD in the contemporary social context might exert a negative impact on the self-construction of individuals involving online gaming behavior. Although Internet gamer is a relatively new social group, a stereotype considering them as unattractive, unpopular, and having poor social skills has already come into being (Kowert et al., 2012). As a member of the group, the perceived stereotype of Internet gamers would affect one’s identity and behavior and would probably result in a higher level of social anxiety. Research in the future can explore how the perceived stereotype influences the association between gaming behavior and social anxiety.

The current study has several limitations. First, the current study was lacking in longitudinal data and could not confirm potentially causal relationships among IGD, social anxiety, and mind-wandering. Therefore, further study is required to conclude a causal inference. Nevertheless, we first provided evidence for the possible correlation between IGD and mind-wandering and replicated the results with an independent sample. Second, the diagnosis of IGD was based only on questionnaires relaying on self-report, which could have resulted in a misclassification for some individuals. Third, the sample included a high proportion of college students, which may hamper some of the findings being generalized to other populations. Finally, the current study only used a simple Mind-Wandering Questionnaire with five items. Although it has been validated across college, high school, and middle school samples (Luo et al., 2016) and the questionnaire measuring mind-wandering was significantly correlated with task-unrelated thought captured by a thought sampling experiment (Mrazek et al., 2013b), further study is warranted to validate the results with multiple instruments to better characterize mind-wandering.

## Conclusion

In conclusion, our study found a positive association between IGD and the level of mind-wandering, and the relationship was partially mediated by social anxiety. While preoccupation of gaming activity may directly increase the occurrence of mind-wandering, IGD may elevate mind-wandering through an indirect path involving social anxiety in which a negative public stereotype of addiction may play an important role. The detrimental consequences of mind-wandering deserve more attention in IGD studies in the future, especially when studying adolescents and young adults in an academic setting.

## Data Availability Statement

The raw data supporting the conclusions of this article will be made available by the authors, without undue reservation.

## Ethics Statement

The studies involving human participants were reviewed and approved by Research Ethics Review Board of Zhejiang University. The patients/participants provided their written informed consent to participate in this study.

## Author Contributions

JZ, YH, XS, and FG designed the study. JZ collected the data. JZ, ZH, and YH analyzed data. JZ and YH drafted the manuscript. FG, ZH, and XS revised the manuscript. All authors contributed to the article and approved the submitted version.

## Conflict of Interest

The authors declare that the research was conducted in the absence of any commercial or financial relationships that could be construed as a potential conflict of interest.

## Footnotes

1 http://www.afhayes.com
